# Low diversity of planktonic bacteria in the tropical ocean

**DOI:** 10.1038/srep19054

**Published:** 2016-01-11

**Authors:** Mathias Milici, Jürgen Tomasch, Melissa L. Wos-Oxley, Hui Wang, Ruy Jáuregui, Amelia Camarinha-Silva, Zhi-Luo Deng, Iris Plumeier, Helge-Ansgar Giebel, Mascha Wurst, Dietmar H. Pieper, Meinhard Simon, Irene Wagner-Döbler

**Affiliations:** 1 Helmholtz-Center for Infection Research, Group Microbial Communication, Braunschweig, Germany; 2 Helmholtz-Center for Infection Research, Group Microbial Interactions and Processes, Braunschweig, Germany; 3 Institute for Chemistry and Biology of the Marine Environment, University of Oldenburg, Oldenburg, Germany

## Abstract

The diversity of macro-organisms increases towards the equator, with almost no exceptions. It is the most conserved biogeographical pattern on earth and is thought to be related to the increase of temperature and productivity in the tropics. The extent and orientation of a latitudinal gradient of marine bacterioplankton diversity is controversial. Here we studied the euphotic zone of the Atlantic Ocean based on a transect covering ~12.000 km from 51°S to 47 °N. Water samples were collected at 26 stations at five depths between 20 and 200 m and sequentially filtered through 8 μm, 3 μm and 0,22 μm filters, resulting in a total of 359 samples. Illumina sequencing of the V5–V6 region of the 16S rRNA gene revealed a clear biogeographic pattern with a double inverted latitudinal gradient. Diversity was higher in mid-latitudinal regions of the Atlantic Ocean and decreased towards the equator. This pattern was conserved for bacteria from all three planktonic size fractions. Diversity showed a non-linear relationship with temperature and was negatively correlated with bacterial cell numbers in the upper depth layers (<100 m). The latitudinal gradients of marine bacterial diversity and the mechanisms that govern them are distinct from those found in macro-organisms.

The planktonic bacteria in the oceans comprise one of the largest and most active microbial communities of the planet^1^. These bacteria contain the enzymatic machineries that drive the global biogeochemical fluxes of the elements for life^2^. They are powering the cycles of carbon, sulfur and nitrogen^3^,^4^,^5^, with important consequences for the composition of greenhouse gases in the atmosphere, algal blooms, and ocean acidification. In view of these global challenges, understanding bacterioplankton biogeography is of utmost importance.

One of the key questions in biogeography is the pattern of diversity. In macro-organisms, diversity peaks in the tropics, an observation which dates back to Alexander von Humboldt^6^. It is the most universal biogeographic pattern on the planet and is found, with very few exceptions, across taxa, habitats, body sizes and functional groups of organisms^7^. It is similarly strong in marine as in terrestrial habitats, and has been demonstrated for marine phytoplankton, protozoa and zooplankton^8^. More than 30 hypotheses have been debated to explain this pattern^9^. The two factors thought to be key are productivity (the larger pie can be divided in more pieces) and temperature (the red queen runs faster when she is hot)^6^,^10^.

Given the intimate symbiotic interactions between prokaryotes and eukaryotes and the importance of understanding the ecological consequences of global warming, it is mandatory to know if the concepts established for macro-organisms hold for bacteria as well. The first study to investigate this question found a decrease of species richness with latitude^11^. Then, an analysis of 103 samples taken around the world and using ARISA (Automated Ribosomal Intergenic Spacer Analysis) to type the community members also found a negative correlation between diversity and latitude; moreover, a positive relationship with temperature was observed, suggesting that global macro-ecological patterns hold for bacteria too and that indeed the kinetics of biological processes might have a strong influence on diversity^6^.

Biogeographic studies in the ocean, which were traditionally hampered by low sampling depth (in relation to the huge dimensions of this ecosystem) and lack of taxonomic resolution, have profited enormously from next generation sequencing and large international sampling efforts (e.g. International Census of Marine Microbes (ICoMM), Census of Antarctic Marine Life (CAML)). It has now become possible to directly observe microbial diversity on a global scale with OTU level taxonomic resolution. However, regarding the pattern of alpha diversity, the results are conflicting. A study of 277 epipelagic samples^12^ covering a range from 74.4 °N to 75.6 °S found a negative correlation between species richness and latitude both in the Northern and the Southern hemisphere, which was week but significant and confirmed the above described investigation^6^. A comparable study^13^ using a smaller sample set but including also winter and summer samples did not find a latitudinal diversity gradient.

To take advantage of the numerous studies that have been undertaken by different groups with different methodologies, a modelling approach was recently applied covering 377 marine samples from 164 locations with depths <150 m^14^. Species distribution modelling (SDM) was used to predict the global distribution of taxa by extrapolating from the available samples and environmental data. This approach robustly predicted (1) an inverted latitudinal diversity gradient and (2) an extreme seasonality of this gradient, such that diversity peaked in the higher latitudes of the Northern hemisphere in winter and in the higher latitudes of the Southern hemisphere in summer. The authors suggest that bacterioplankton biogeography follows different rules than those found for macro-organisms. Accordingly there was no overlap between diversity hotspots for bacteria and macro-organisms predicted in this study^14^.

Thus, it is still controversial if bacterioplankton diversity peaks in the tropics or in higher latitudes. The few studies that were undertaken do not provide consistent results. To directly address this important question, here we use a spatially highly resolved sample set from the Atlantic Ocean. The samples were obtained during a 5 week transect (10 April – 15 May 2012) and span a geographical distance of ~12.000 km (51 °S to 47 °N) (Fig. 1). For each of the 26 stations, 5 epipelagic depths between 20–200 m were sampled, and each sample was divided into three size classes by sequential filtering. Altogether 359 samples were obtained. Deep sequencing of the V5–V6 region of the 16S rRNA gene was carried out using Illumina amplicon sequencing. Thus we can clearly demonstrate the global patterns of species richness of the free-living and particle associated planktonic bacteria in the Atlantic Ocean. We then investigated the correlation of microbial diversity with temperature and, as a proxy of biomass, bacterial cell numbers, to determine if the mechanisms acting on macro-organisms are also shaping diversity patterns in marine bacteria.

## Results

### Overview of sequencing results

More than 12 million reads were obtained after quality control. Of those, roughly 10 million reads were affiliated to Bacteria and entered the OTU definition pipeline (Table S1). The average number of bacterial sequences per sample was similar for the three different communities: FL (free-living bacteria, 0.22-3 µm) 27,595 ± 12,974, SPA (small particle associated bacteria, 3-8 µm) 33,230 ± 10,766 and LPA (large particle associated bacteria > 8 µm) 26,858 ± 12,711 (Table S1). A total of 259 OTUs were obtained for the bacterial sequences from the 0.22 μm filter (FL), 269 OTUs for the bacterial sequences from the 3 μm filter (SPA) and 236 OTUs for the bacterial sequences from the 8 μm filter (LPA) (Table S1). Rarefaction analysis after resampling to 6456 sequences per sample showed that a plateau of saturation was reached for all three filters, indicating good coverage of bacterial diversity (Figure S1).

### Latitudinal and depth distribution of bacterial diversity

**Figure 2:**
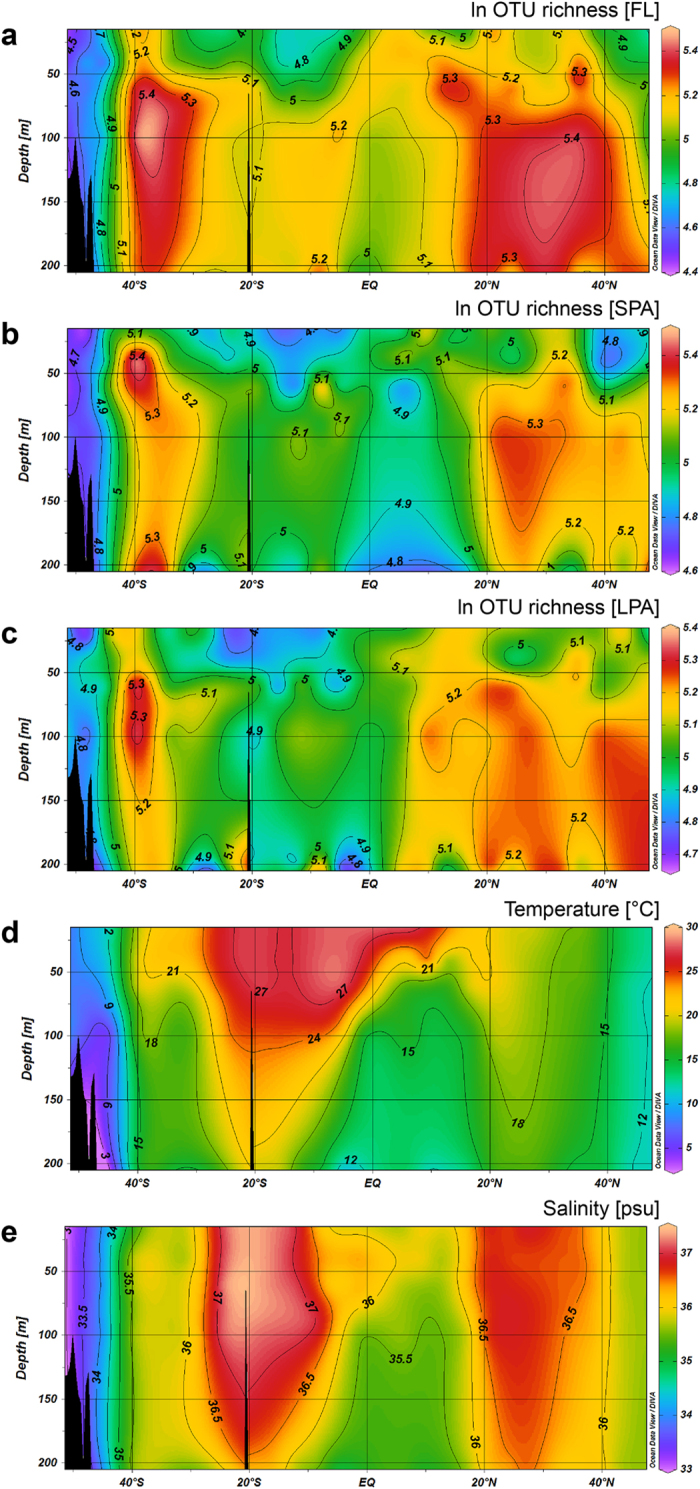
Bacterial diversity, water temperature and salinity in the epipelagic zone of the Atlantic Ocean. Bacterial diversity is shown as the ln of OTU richness (S) from 20 to 200 m depth from 51 °S to 47 °N for the three size fractions of the marine plankton: FL (free living bacteria, 3–0.22 μm filtrate) (**a**), SPA (small particle associated bacteria, 8–3 μm filtrate) (**b**) and LPA (large particle associated bacteria, >8 μm filtrate) (**c**). Panel (**d**) shows the water temperature and (**e**) the salinity. The five sections were generated with the free software Ocean Data View (Schlitzer, R., Ocean Data View, http://odv.awi.de, 2015), version 4.7.2, in accordance with the metadata reported in Supplementary Table S2.

Bacterial diversity showed a nonlinear relationship with latitude (Fig. 2). Moving from both sides of the transect, North and South hemisphere exhibited a similar pattern. Bacterial diversity showed an increase in diversity towards the subtropical regions of the Atlantic Ocean (20-40° absolute latitude) with a subsequent decrease towards the equator (20°-0° absolute latitude). While the absolute values of diversity differed in the various depths across the epipelagic zone, similar latitudinal patterns were found at all depths. Those patterns were consistent for both hemispheres and for bacteria from all three size fractions of the marine plankton. However, the South hemisphere was less diverse than the North, and the peak of diversity for the South Atlantic covered a narrower latitudinal range (30–40 °S) compared to the North Atlantic (20–40 °N).

**Figure 3:**
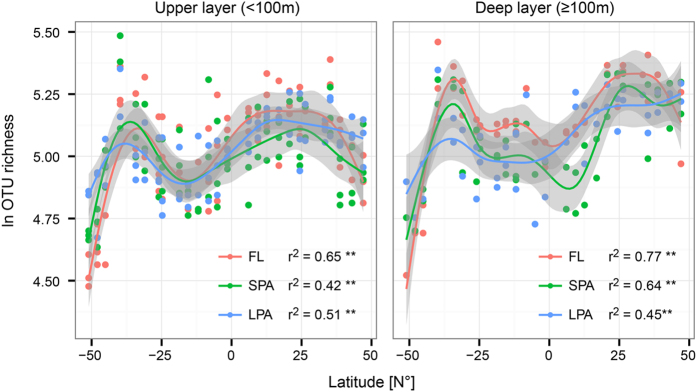
Latitudinal course of bacterial diversity. The OTU richness (ln S) at each sampling site and depth was plotted against the latitude for the upper (>100 m, left) and lower (>100 m, right) water depths. Generalized Additive Models were fitted with cubic spline. Color code indicates the size fraction of the marine plankton. The coefficient of determination (adjusted R^2^) and its significance (**p < 0.01) are reported for each model.

Using a generalized additive model with cubic spline, we could build a separate model for bacteria from each of the three size fractions of the marine plankton, analyzing the upper depths (20 m, 40 m, 60 m) separately from the lower depths (100 m, 200 m) (Fig. 3). All models were highly significant (p value < 0.01), and predicted the latitudinal distribution of diversity across the Atlantic Ocean with high accuracy, with adjusted r^2^ values ranging from 0.42 to 0.77. The highest coefficient of determination was found for the bacteria of the FL community, which r^2^ of 0.65 and 0.77 for the upper and deeper part of the water column, respectively. The bacteria from the particle associated plankton communities SPA and LPA had lower adjusted r^2^ values (between 0.42 and 0.64). The comparison between the upper and deeper part of the water column clearly shows that the pattern described here is depth independent. However, the deeper layers of the epipelagic zone (≥100 m) are more diverse than the upper layers (<100 m).

The data show that bacterial diversity does not peak around the equator. By contrast, the highest diversity is observed in the mid-latitude regions of both hemispheres. This pattern is independent of depth and consistent across the epipelagic zone.

### Effect of salinity and primary productivity on alpha-diversity

The transect analyzed here spanned oceanic regions with salinity concentrations ranging from 33 PSU up to 37.5 PSU. Despite the large gradient of salinity sampled no relationship was observed between bacterial diversity (S), evenness (J‘) and salinity (Fig. 2). In the Northern hemisphere, a broad peak in salinity was found between 20 °N and 30° N across all depths of the epipelagic zone; it partly overlapped with the diversity peak that occurred at the same latitudinal range, but only below 50 m. In the Southern hemisphere, the peaks of diversity and salinity did not overlap; diversity peaked between 30 °S and 40 °S, while salinity peaked between 25 °S and 10 °S. This suggests a nonlinear relationship between diversity and salinity and shows that the pattern of alpha-diversity is not caused by salinity.

Natural fluorescence of chlorophyll can be used as proxy of primary productivity^15^. Therefore we used the fluorescence data from the CTD to show the relationship between bacterial diversity and potential primary productivity (Fig. S2). Fluorescence values were very low throughout the transect. Two regions had relatively high fluorescence values (above 0.4 V). They were both located in the North hemisphere in the upper water layers. The highest values were recorded at 40 °N and ∼5 °N around 50 m, where microbial diversity was intermediate. Overall, there was no direct or inverse relationship between fluorescence and OTU richness. Therefore it seems unlikely that the observed diversity patterns were controlled primarily by productivity.

### Effect of temperature on species richness and species evenness

**Figure 4:**
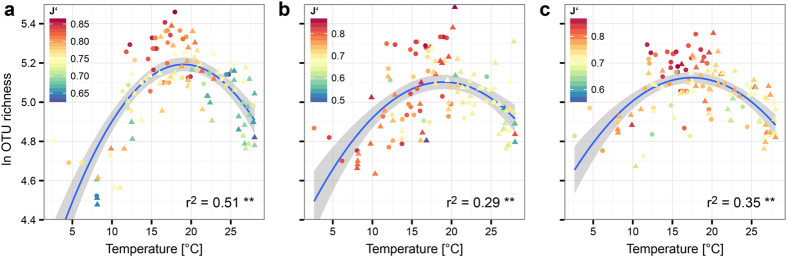
Relationship between OTU richness and temperature. Bacterial diversity expressed as the ln of OTU richness (S), was plotted against the water temperature. A second order polynomial model was fitted to the data and significance was calculated for permutations (999). A separate model was constructed for each of the size fractions of the marine plankton: FL (**a**), SPA (**b**) and LPA (**c**). All three models were highly statistically significant (** p < 0.01).The color key shows Pielou’s index of evenness (J‘). The coefficient of determination (adjusted R^2^) is reported for each of the models in the upper right part of the graphs. Triangles represent samples from depths above 100 m, while circles represent samples from depths >100 m.

Bacterial diversity showed a nonlinear relationship with temperature which was independent from depth (Fig. 4). The data could be modelled with high statistical significance (p value < 0.01) by a second order polynomial distribution. For the free-living bacterioplankton (FL), the adjusted r^2^ value was 0.51, while for the small and large particle associated bacteria (SPA and LPA) it was slightly lower (0.29 and 0.35, respectively). Bacterial diversity was lowest at low temperatures ( < 10 °C). The highest diversity was found between 15–20 °C, and above 20 °C diversity decreased again. The data suggest that most marine bacterial species are adapted to temperatures between 15–20 °C. Moreover those communities were not only more diverse, but they exhibited a more even distribution of species abundance as observed using Pielou’s index of evenness (J‘). The high diversity in the mid-temperature range from 15–20 °C was coupled with a high evenness of the community, while at the two extremes of the temperature curves both evenness and diversity were much lower. This was especially pronounced for the free-living and large particle associated bacterial communities. Our data show that in the Atlantic Ocean species diversity and evenness do not increase with temperature in a linear way. By contrast, both peak around 15–20 °C.

### Bacterioplankton diversity and cell numbers

**Figure 5:**
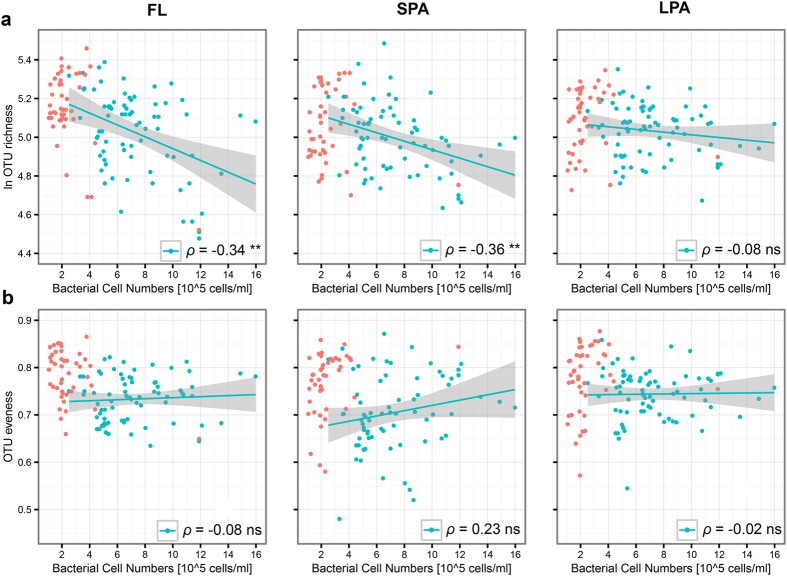
Relationship between alpha diversity and bacterial cell numbers. OTU richness (S), and bacterial evenness (J‘) were correlated with cell numbers (cells/ml) for the depths above 100 m. Spearman rank correlation with 999 permutations was calculated. Significance level (**p < 0.01, *p < 0.05 and ns p > 0.05) and Rho values (*ρ*) are shown on the chart area. (a) Correlation for the three separated communities: FL, SPA and LPA between OTU richness (S) and cell numbers (cells/ml). (**c**) Correlation for the three separated communities: FL, SPA and LPA between OTU evenness (J‘) and cell numbers (cells/ml). Grey shading shows 95% confidence intervals for Spearman rank correlation. Red circles show data for depths >100 m. Here, cell numbers were low and had no correlation with diversity.

Bacterial diversity (S) was negatively correlated with cell numbers for the FL (ρ = −0.34) and SPA (ρ = −0.36) microbial communities in the upper layers (<100 m) of the epipelagic zone (Fig. 5A). Bacterial evenness did not show any significant correlation with cell numbers (Fig. 5B). For the lower depths of the water column (≥100 m) no correlation between alpha diversity indices and bacterial cell numbers was found. The data for the deep samples are shown in Fig. 5 as red dots, but a regression line was not calculated. These samples had a very low cell density (Fig. S2) and a relatively high diversity (Fig. 2).

Thus, in the upper 100 m of the epipelagic zone, at high cell numbers, free-living or small particle associated microbial communities had a smaller number of species.

## Discussion

We found a clear geographic pattern for bacterial diversity in the epipelagic zone of the Atlantic Ocean. It showed a double inverted latitudinal gradient and peaked between 40° and 20° absolute latitude. The data are in contrast to the negative or lacking correlations with latitude described previously^6^,^11^,^12^,^13^. They confirm the modelling study of Ladau *et al.*^14^ with respect to the summer peak of diversity in the Southern ocean at 30–40 °S, which we found, too. However, we at the same time found a diversity peak in the Northern hemisphere at about the same absolute latitude. Thus we can conclude that diversity peaks at intermediate absolute latitudes independent from the season. Marine bacteria clearly do not follow the macro-ecological pattern of increased diversity in the tropics, which is an intriguing finding in biogeography. Although they share their spatial niche with eukaryotic microalgae and protozoa and widely interact with them^16^, their patterns in diversity are apparently not directly correlated with those of the other members of the biota.

The maximum of both diversity and evenness was found in a temperature range from 15 °C to 20 °C, with lower diversity both above and below those temperatures. Thus, there was no linear correlation with temperature, but a bell-shaped curve. This suggests that parameters other than temperature control bacterial diversity. They, of all organisms, are the smallest free-living cells with no means to buffer the linear effect of temperature on biological processes, but their global diversity is not increased at the temperatures found in the tropics, rejecting the “red queen runs faster when she is hot” hypothesis for bacteria. In a gigantic metagenome study of the global ocean microbiome Sunagawa *et al.*^17^ observed a similar pattern, with richness showing a maximum around 15 °C and declining below and above. Here, also, richness was highest in mid-latitudinal ranges rather than towards the equator. Our data show that this pattern is consistently found not only for free-living bacteria, but also for those living in the large and small particle associated fractions of the ocean plankton. Thus, in contrast to macro-organisms, bacterial communities do not show an increase in diversity with temperature. The underlying mechanisms appear to be acting globally and thus must be highly conserved. The strong seasonality of marine communities would make time-series data from oceanographic stations an ideal dataset to analyze them in depth^18^.

Our data show that in the upper depth layers of the epipelagic zone the diversity of free-living and small particle associated bacteria (FL and SPA) negatively correlates with bacterial cell numbers. Could this be caused by top-down control through phages ? In the surface ocean, the abundance of free-living bacteria is maintained at ∼5 × 10^5 ^cells/ml by the combined action of bacteriophages and protozoan grazing^19^. An increase in the abundance and diversity of bacteriophages would be expected to increase microbial diversity, yet a recent metagenomics study did not find any pattern in diversity for marine viruses^20^, while previously a higher diversity of viruses was found in the tropics^19^. Thus it seems unlikely that bacteriophages could be responsible for the observed decrease in diversity at higher cell numbers.

Could the smaller diversity in higher biomass samples be caused by different growth strategies at low and high nutrient concentrations ? In macro-ecological theory the concept of r- and K-selection was developed to describe the evolution of different life-history strategies; briefly, selection for fast growth rates (r-selection) was hypothesized to favor small body size, high productivity, and variable population size, while selection for high population size (K-selection, derived from the term for carrying capacity of an ecosystem) was hypothesized to favor slow growth rate, strong competitive abilities, stable population size and high efficiency with respect to resource utilization^21^. An increase in r-selection within a community due to disturbance can result in reduced diversity^22^. In microbial ecology, this paradigm was replaced by the concept of trophic strategy^23^. Copiotrophs (r-strategists) are fast growing bacteria adapted to high concentrations of nutrients; they represent the majority of bacteria that can be cultivated in the laboratory. Oligotrophs (K-strategists), by contrast, are slow growing bacteria adapted to low substrate concentrations; in the ocean, they represent the dominant populations and are characterized by streamlined genomes^24^,^25^,^26^. Thus, the shift in diversity observed in the higher biomass bacterioplankton communities might indicate that these communities responded to transient changes in growth conditions, like nutrient import, algae blooms etc. by overgrowth of copiotrophs. Interestingly, a study of an algal bloom in the North Sea discovered a decrease of OTU richness in the bloom area^27^, in full accordance with this hypothesis. In a mesocosm experiment a loss of rare OTUs was found upon experimental fertilization^28^. During a bloom of the dinoflagellate *Akashiwo sanguine* in the Xiamen Sea in China the diversity of free-living bacteria was also clearly reduced compared to the control area^29^. An in-depth analysis of the seasonal succession of microbial populations in the North Sea in spring discovered a reduction in Shannon diversity as well as evenness during an algal bloom^30^.

In conclusion we here demonstrate that bacterial alpha-diversity does not follow a negative latitudinal gradient in marine bacterioplankton. Bacterial richness (S) and bacterial evenness (J‘) is higher in mid-latitude regions of the Atlantic Ocean with a resulting double inverted latitudinal gradient. Parameters expected to shape biogeographic diversity patterns like salinity, fluorescence, temperature and biomass (cell numbers) do not show a positive linear relationship with diversity, but rather a negative relationship (cell numbers), a highly nonlinear relationship (temperature) or no effect (salinity and fluorescence). Thus we can conclude that not only the latitudinal diversity patterns of bacteria are completely different from those of macro-organisms, also the mechanisms shaping these patterns appear to be different: Neither is the larger pie divided into more pieces, nor does the hot queen run faster.

It is of course not possible to causally explain these findings based on a descriptive dataset and correlation analyses, and moreover, decades of research in macro-ecology also have not yet provided a definite solution for the most conspicuous diversity pattern on the planet. One plausible, yet wildly speculative, hypothesis would be that a larger diversity of marine bacterial communities might be indicative of an undisturbed community close to its carrying capacity. This would be in accordance with the higher diversity in deeper samples^17^ and in the center of oceanic gyres compared to the edges. These gyres are the most oligotrophic regions of the ocean; their center is very stable and practically devoid of nutrients (hyper-oligotrophic), yet the diversity of Bacteria and Archaea is higher in the center 31. However, that would mean that the peaks in diversity in the Northern and Southern subtropical regions reflect stable communities, while surface samples and those around the equator are subject to disturbances, e.g. turbulences at the edges of oceanic gyres, the influence of upwelling deep waters, or others, which is counterintuitive and not supported by the data that we have. Alternatively, the peaks in diversity might be located in areas where two different habitats (defined e.g. by salinity, temperature, nutrients etc.) overlap, such that species from both habitats are found at the intermediate zone. Unraveling those mechanisms will be important for understanding the response of the marine microbiome to global warming.

## Materials and Methods

### Sampling

Samples were collected during cruise ANT-28/5 (10 April – 15 May 2012 with RV Polarstern) at 26 stations across a latitudinal transect in the Atlantic Ocean (51 °S–47 °N). At all stations, samples were consistently collected from five depths of the epipelagic zone: 20 m, 40 m, 60 m, 100 m and 200 m. Seven samples were taken ±10 m from the designated depths (Table S2). Sampling was carried out with 12 L Niskin bottles mounted on a CTD probe (Sea-Bird Electronics Inc. SBE 911 plus probe) equipped with double temperature and conductivity sensors, a pressure sensor, altimeter, chlorophyll fluorometer and transmissometer. CTD data were validated during the cruise through regular reference measurements of water samples applying standard methods. Immediately after retrieval on deck, 12 L of sample water from one bottle was transferred to 20 L wide-mouth barrels, and filtered with three peristaltic pumps (Ismatec, IDEX Health & Science GmbH, Wertheim Germany) through three successive stainless steel filtration devices (Druckfiltrationsgerät Edelstahl Typ 1627, Omnilab Laborzentrum, Braunschweig, Germany) equipped with the following membrane filters (diameter 142 mm): 8 μm (mixed cellulose ester SCWP14250, Millipore, Darmstadt, Germany), 3 μm (mixed cellulose ester SSWP14250, Millipore, Darmstadt, Germany), and 0.22 μm (polyethersulfone GPWP14250, Millipore, Darmstadt, Germany). After filtration membranes were immediately stored at −80 °C until DNA isolation. The three communities were named: free-living (FL) for the 0.22 μm membranes, small particle associated (SPA) for the 3 μm membranes and large particle associated (LPA) for the 8 μm membranes (Supplementary Table S2).

### Cell counts

The determination of cell numbers of the free-living microorganisms was conducted with a BD Accuri C6 cytometer (BD Biosciences, USA) using SybrGreen I (Invitrogen, United Kingdom) after staining and the internal fluidics calibration of the device. The fixed samples (2% final concentration glutardialdehyde, Carl Roth, Germany) were filtered through 50 μm filters (Cell Trics, Partec) to remove larger particles. Volume verification was done using TruCount beads (BD) as described previously^32^,^33^.

### DNA isolation

Total DNA was isolated with a combined chemical and mechanical procedure using the UltraClean Soil DNA Isolation KIT (MO BIO Laboratories, Inc., Carlsbad, CA, USA) with modifications. Approximately 1/8 of each filter was rapidly retrieved and cut in slices that were transferred into a 2 ml tube containing garnet beads (0.70 mm, MO-BIO Laboratories Inc., Carlsbad, CA, USA), 60 μl of solution S1 and 200 μl of solution IRS (MO-BIO Laboratories Inc., Carlsbad, CA, USA) and 800 μl of a lysis buffer (as in 34 but excluding CTAB). Tubes containing the cellulose acetate filters (3 μm and 8 μm) were pre-treated by bead beating to fragment the filter (1 min, 6 m/s) using the Ribolyser (MP Biomedicals, OH, USA). The polycarbonate filters (0.22 μm) were rigid enough to be cut in small stripes that were easily accessible by the beads in the vortex adapter and thus bead-beating was not necessary for them, as determined in preliminary experiments. The tubes were fixed horizontally to the vortex adapter (MO BIO Laboratories, Inc., Carlsbad, CA, USA) and shaken for one hour for mechanical disruption of bacteria. Immediately afterwards samples were incubated for 20 min with 25 μl proteinase K (22 mg/ml) at 37 °C. The tubes were then centrifuged as recommended by the manufacturer and the lysate was collected in a fresh tube. The sample was centrifuged again and the lysates were combined. The extraction was continued according to the manufacturer’s instructions, with the exception of the precipitation time of the DNA, which was prolonged to 1 hour. The DNA was solved in 20 μl of autoclaved distilled H2O and stored at −20 °C. Except for the bead-beating, all filters were treated equally. High molecular weight DNA was obtained from both the cellulose acetate and the polycarbonate filters.

### Illumina sequencing and bioinformatics analysis

Library preparation was performed according to 35,36. For Illumina sequencing we used primers 807F and 1050R for the hypervariable V5–V6 region^37^. Libraries were sequenced using 250 bp paired-end sequencing chemistry on an Illumina MiSeq platform. A total of 25,254,193 raw sequence reads were obtained. The raw data were submitted to the ENA (European Nucleotide Archive) database and were assigned the BioProject ID: PRJEB11493. Quality filtering and definition of OTUs were performed as described^35^. Briefly, first raw reads were quality trimmed. By sliding a window of 10% of the read length along the sequence the average quality was assessed. Read fragments from the 3’ end of the reads that had a PHRED score of the fastq file (Q value) below 10 were removed. Reads that after trimming were shorter than 140 nt, had an N character in their sequence, any mismatches within the primers and barcodes or more than 10 homopolymer stretches were removed. Next, reads were demultiplexed and sorted. Reads were trimmed conservatively to 140 nt. The paired ends were subsequently matched to give 280 nt. The Mothur program unique.seqs was used to collapse the reads. The paired-end reads (140 nt) of each OTU were merged to fully cover the V5–V6 region with EMBOSS merger (http://emboss.bioinformatics.nl/cgi-bin/emboss/merger), no mismatch was allowed in the overlapping of the forward and reverse reads and the length of the retrieved sequences was ranging from 238 to 259 nt. OTUs were defined at a level of identity of >99% (two or less mismatches over the whole sequence).

The dataset was then filtered to consider only those OTUs that were present in an abundance >0.001% of the whole experiment and (i) were present in at least one sample at a relative abundance >1% of the total sequences of that sample or (ii) were present in at least 2% of samples at a relative abundance >0.1% for a given sample, or (iii) were present in at least 5% of samples at any abundance level. The OTUs that passed these thresholds were used as the basis for all subsequent analyses. To test the reproducibility of our sequencing approach 19 samples were sequenced twice (Figure S3). Pearson correlation between replicates was between 0.92 and 0.99 (Figure S3).

### Data analysis

Taxonomic classification was assigned using SINA aligner (version 1.2.11)^38^ employing the reference database SILVA (119 NR)^39^. The OTUs were aligned and classified against a maximum of 100 sequences that had a minimum of 97% similarity with the query sequence, using the lowest common ancestor method (LCA). All OTUs that were not assigned to the domain bacteria were excluded from the analysis. Number of sequences per sample was homogenized via resampling to 6456 with the statistical program R (http://www.Rproject.org/, v. 3.0.1) with the library vegan: Community Ecology Package (v. 2.0–8). Alpha diversity indexes were calculated in Primer 6 (v.6.1.6, PRIMER-E, Plymouth Marine Laboratory, Plymouth, UK; Clarke and Warwick, 2001) based on the number of OTUs (S) referred as OTU richness and with the Pielou´s index of evenness J‘. Data representation was performed with ocean data view version 4.7.2 (Schlitzer, R., Ocean Data View, http://odv.awi.de, 2015). Modelling and Spearman rank correlations were carried out with the statistical program R (http://www.Rproject.org/, v. 3.0.1). In both cases significance level was tested via permutation (999). For modelling, the “Generalized Additive Models” (GAM) function with cubic spline was used in R.

## Additional Information

**How to cite this article**: Milici, M. *et al.* Low diversity of planktonic bacteria in the tropical ocean. *Sci. Rep.* **6**, 19054; doi: 10.1038/srep19054 (2016).

## Supplementary Material

Supplementary Information

Supplementary Table S1

Supplementary Table S2

## Figures and Tables

**Figure 1:**
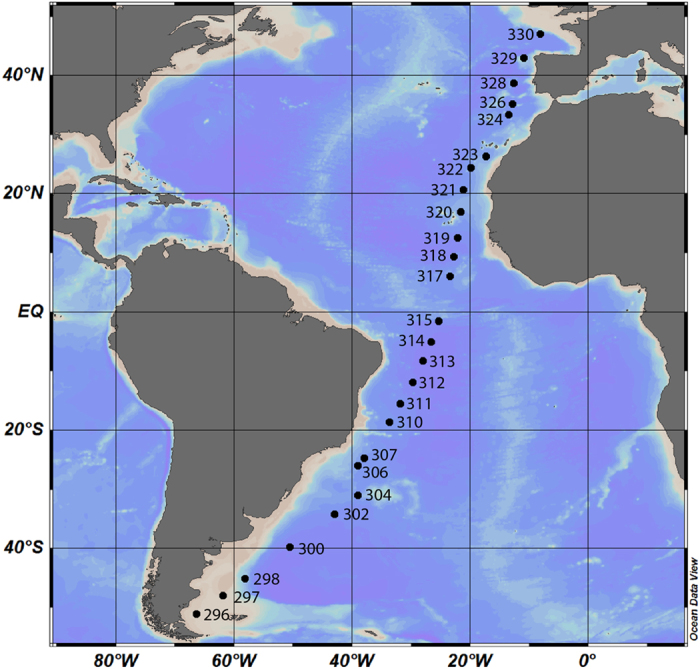
Sampling stations of cruise ANT 28-5 across the Atlantic Ocean. At each station, the epipelagic zone was sampled from 20 to 200 m. The free software Ocean Data View (Schlitzer, R., Ocean Data View, http://odv.awi.de, 2015), version 4.7.2, was used to generate the map in accordance with the geographical position reported in Supplementary Table S1.
