# Supplementary material for: Low diversity of planktonic bacteria in the tropical ocean
Source: Sci Rep. 2016 Jan 11;6:19054. doi: 10.1038/srep19054 (PMC4707477; doi:10.1038/srep19054)
Supplement: Supplementary Information [file srep19054-s1.pdf]

Supplementary Material

Low diversity of planktonic bacteria in the tropical ocean

Mathias Milici[1*], Jürgen Tomasch[1], Melissa L. Wos-Oxley[2], Hui Wang[1], Ruy Jáuregui[2], Amelia Camarinha-Silva[2$], Zhi-Luo Deng[1], Iris Plumeier[2], Helge-Ansgar Giebel[3], Mascha Wurst[3], Dietmar H. Pieper[2], Meinhard Simon[3] and Irene Wagner-Döbler[1]

[1]Helmholtz-Center for Infection Research, Group Microbial Communication, Braunschweig, Germany

[2]Helmholtz-Center for Infection Research, Group Microbial Interactions and Processes, Braunschweig, Germany

[3]Institute for Chemistry and Biology of the Marine Environment, University of Oldenburg, Oldenburg, Germany

[$]Current address: University of Hohenheim, Institute of Animal Science, Stuttgart, Germany

*Corresponding author: Mathias Milici, Helmholtz-Center for Infection Research, Inhoffenstr. 7, 38124 Braunschweig. Email Mathias.Milici@helmholtz-hzi.de; phone 0049-531-61813082.

Running title: Alpha diversity of marine bacterioplankton

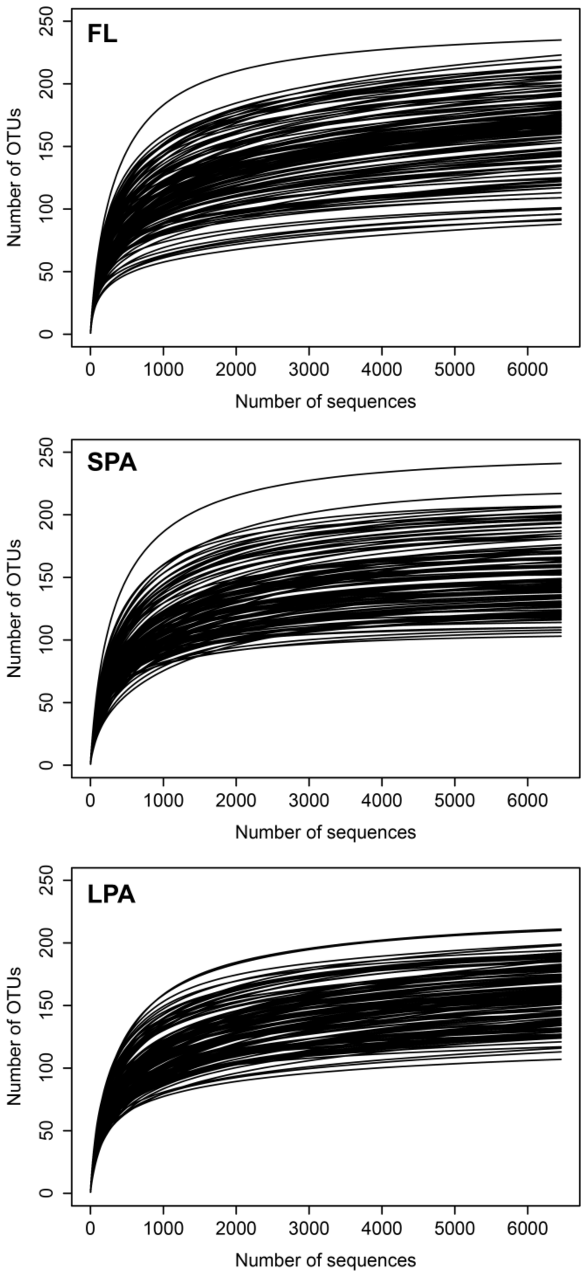

Supplementary Figure S1: Rarefaction analysis: Rarefaction analysis was performed for bacterial reads from each size fraction of the plankton. Plots show (from top to bottom) rarefaction for the FL, SPA and LPA communities after resampling to 6456 sequences.

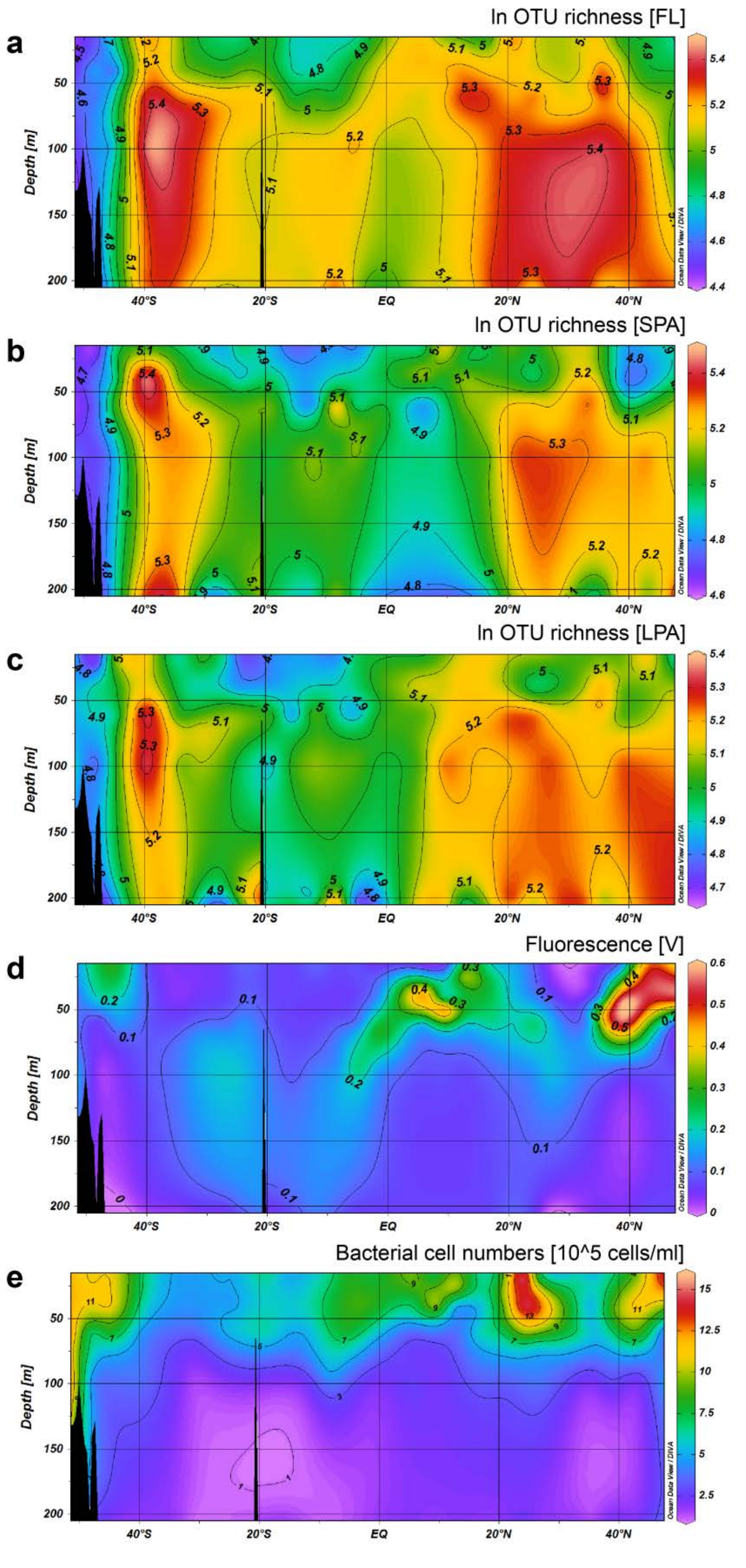
a
ln OTU richness [FL]
b
ln OTU richness [SPA]
c
ln OTU richness [LPA]
d
Fluorescence [V]
e
Bacterial cell numbers [10^5 cells/ml]
Depth [m]
40°S
20°S
EQ
20°N
40°N
Ocean Data View / DIVA

Supplementary Figure S2: Bacterial diversity, fluorescence and cell numbers in the epipelagic zone of the Atlantic Ocean: Bacterial diversity is shown as the ln of OTU richness (S) from 20 to 200 m depth from 51°S to 47°N for the three size fractions of the marine plankton: FL (free living bacteria, 3 – 0.22 µm filtrate) (a), SPA (small particle associated bacteria, 8 – 3 µm filtrate) (b) and LPA (large particle associated bacteria, >8µm filtrate) (c). Panel (d) shows the fluorescence measured *in situ* and (e) shows cell density. The five sections were generated with the free software Ocean Data View (Schlitzer, R., Ocean Data View, http://odv.awi.de, 2015), version 4.7.2, based on the metadata in Table S2.

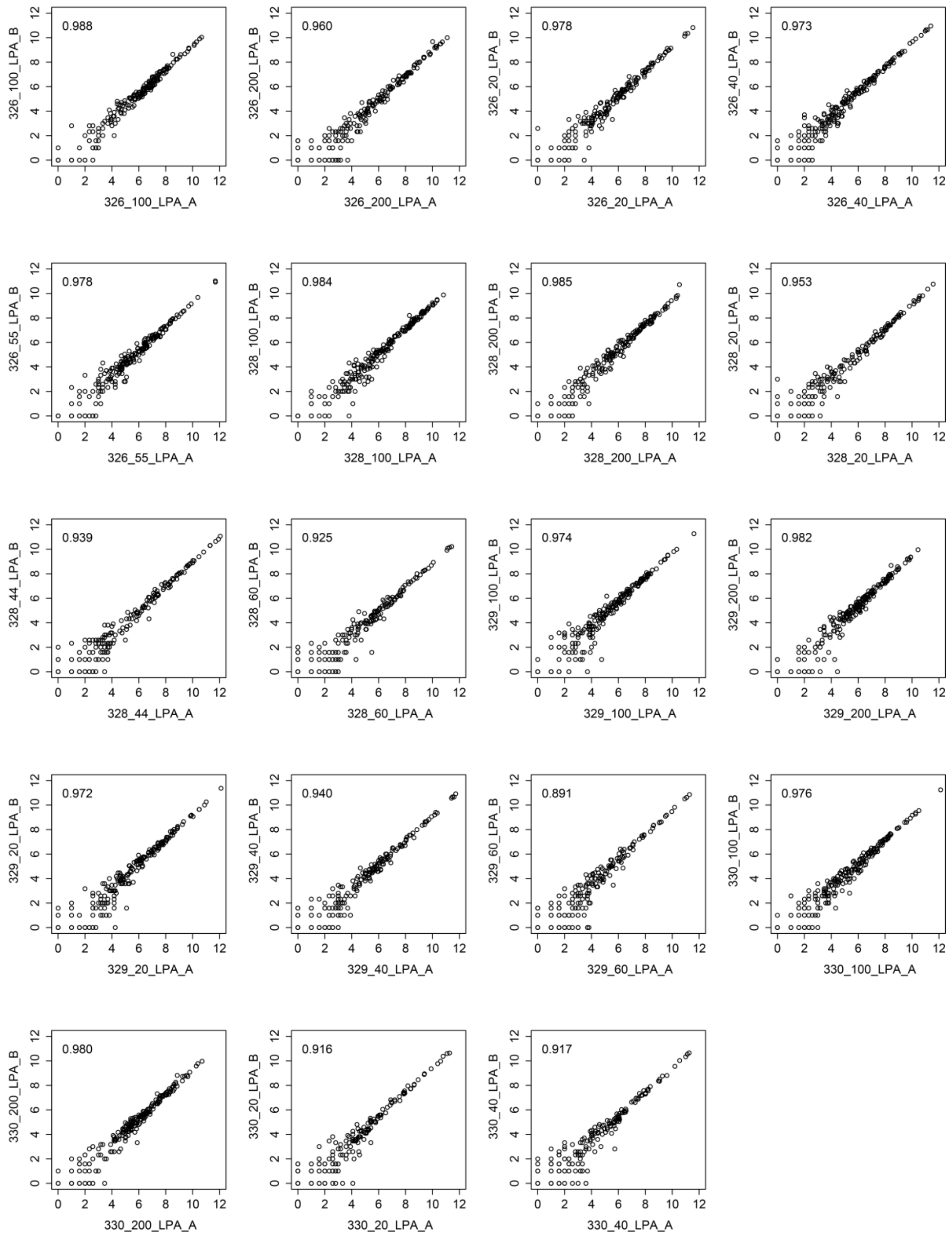

Supplementary Figure S3: Correlation between technical replicates: Pearson correlation was calculated on Log_2 transformed data for 19 samples that were sequenced twice. Correlation values are displayed in the left upper corner of each graph.

Supplementary Table S1: Sequencing information and OTU lists: Sheet 1: Number of raw reads, number of reads after the bioinformatics analysis, number of bacterial reads for each sample. **Sheet 2**: List of OTUs from the FL size fraction, abundance and sequence. **Sheet 3**: List of OTUs from the SPA size fraction, abundance and sequence. **Sheet 4**: List of OTUs from the LPA size fraction, abundance and sequence.

Supplementary Table S2: Environmental parameters and diversity indices. For each sample the sampling depth, temperature, fluorescence, salinity, geographical location and cell number are listed. Additionally, indices of diversity (ln S and J`) are provided for the bacteria from the three size fractions of the marine plankton (FL, SPA and LPA).
